# Automatic Liver Segmentation in CT Images with Enhanced GAN and Mask Region-Based CNN Architectures

**DOI:** 10.1155/2021/9956983

**Published:** 2021-12-16

**Authors:** Xiaoqin Wei, Xiaowen Chen, Ce Lai, Yuanzhong Zhu, Hanfeng Yang, Yong Du

**Affiliations:** ^1^School of Medical Imaging, North Sichuan Medical College, Nanchong, Sichuan 637000, China; ^2^Department of Radiology, Affiliated Hospital of North Sichuan Medical College, Nanchong, Sichuan 637000, China

## Abstract

Liver image segmentation has been increasingly employed for key medical purposes, including liver functional assessment, disease diagnosis, and treatment. In this work, we introduce a liver image segmentation method based on generative adversarial networks (GANs) and mask region-based convolutional neural networks (Mask R-CNN). Firstly, since most resulting images have noisy features, we further explored the combination of Mask R-CNN and GANs in order to enhance the pixel-wise classification. Secondly, *k*-means clustering was used to lock the image aspect ratio, in order to get more essential anchors which can help boost the segmentation performance. Finally, we proposed a GAN Mask R-CNN algorithm which achieved superior performance in comparison with the conventional Mask R-CNN, Mask-CNN, and *k*-means algorithms in terms of the Dice similarity coefficient (DSC) and the MICCAI metrics. The proposed algorithm also achieved superior performance in comparison with ten state-of-the-art algorithms in terms of six Boolean indicators. We hope that our work can be effectively used to optimize the segmentation and classification of liver anomalies.

## 1. Introduction

Segmentation of computed-tomography (CT) liver images is currently a standard technique for computer-assisted diagnosis and therapy, which enjoys the advantages of high accessibility, acceptable acquisition time, and good spatial resolution [1, 2]. The correct estimation of the liver volume (technically referred to as liver volumetry) is an essential prelude and preparation for hepatic surgery that must precede any major hepatectomy or liver transplantation [3, 4]. Currently, there is a growing interest in performing liver volumetry in order to cope with the recent increase in extended hepatectomies, split-liver transplantations, and liver transplantations from living donors [5]. Liver volumetry has been partially or fully automated so as to improve repeatability and accuracy as well as to reduce processing times [6]. However, liver segmentation suffers from a variety of problems and difficulties [7]. Firstly, the segmentation performance is typically degraded by the influence of complex surrounding blood vessels and organs [8]. Also, the liver shape shows high variability across different sections in the same set of CT images [9, 10]. In addition, the density of the liver tissues is highly similar to the densities of many other types of soft tissues in the abdominal cavity [11]. Moreover, medical CT imaging often produces images of low contrast and uneven grey scales, making it difficult to accurately segment liver images [12]. In short, liver segmentation in CT images has become a major challenge as it can hardly achieve the desired or expected outcomes. Numerous deep-learning liver segmentation methods have been proposed in order to alleviate or partially solve the above problems [13, 14]. Such methods can help the radiology staff to further improve liver disease diagnosis, achieve timely detection and treatment, and reduce the death risk due to liver cancer [15].

Although automated segmentation methods have been frequently proposed, those methods have not necessarily been implemented in routine clinical use [16]. The cause of slow adaptation to automation by the medical community is believed to arise from limitations in clinical validation, rather than to stem from lack of technical ingenuity. Most CT images contain fuzzy or noisy features, which can lead to a substantial reduction in segmentation accuracy [17, 18]. An effective automated segmentation method should be equipped with a validation framework that encompasses the following components: (a) employment of a valid reference standard; (b) validation datasets that reflect the actual clinical practice; (c) clear metrics that assess and measure the segmentation precision, accuracy, efficiency, and error; and (d) a comparison of the aforementioned metrics via agreed-upon effective statistical tools.

We further explored the combination of Mask R-CNN and GANs to enhance the pixel-wise classification performance. Also, *k*-means clustering was used to lock the image aspect ratio in order to get more essential anchors which can help boost the liver segmentation performance in computed tomography (CT) images. Indeed, the segmentation process is quite challenging and crucial due to the fuzziness of the liver pixel boundaries, the highly similar intensity patterns of the liver and its neighbouring organs, the high noise levels, and the large variations in tumor shape and appearance. Therefore, liver segmentation in CT images should be effectively performed before other tasks of target measurement, detection, and recognition. A GAN [19, 20] is also integrated in the mask region-based convolutional neural network (Mask R-CNN) architecture in order to create a new GAN-Mask R-CNN framework that boosts the liver segmentation performance in CT images [21]. In our framework, we make four key contributions:

1. We explored pixel-wise classification enhancements through the combination of Mask R-CNN and GANs, augmentation of the Mask R-CNN training data, and exploitation of the generated synthetic data
2. We used *k*-means clustering to lock the image aspect ratio in order to get more key anchors which can help get better segmentation results
3. The performance of the proposed framework was compared against that of the conventional Mask R-CNN algorithm, in terms of the Dice similarity coefficient (DSC), volume overlap error (VOE), relative volume difference (RVD), the average symmetric surface distance (ASSD), root-mean-square symmetric surface distance (RMSD), and maximum symmetric surface distance (MSSD)
4. Additionally, our proposed GAN Mask R-CNN achieved superior performance in comparison with ten state-of-the-art algorithms. The comparison is based on the other six indicators including the overall accuracy, sensitivity, specificity, precision, false discovery rate (FDR), and false omission rate (FOR)

## 2. Related Work

Accurate diagnosis is highly required for liver therapy planning, liver size evaluation, and optimal clinical decision-making. Medical CT imaging provides accurate anatomical information for the human abdominal organs, especially liver segmentation and disease diagnosis [22]. Also, liver anatomy visualization and segmentation from CT scans provide significant guidance for liver surgery planning. For CT-based clinical diagnosis of liver diseases, reliable and accurate liver segmentation and identification of surrounding anatomical structures are crucial for subsequent treatment planning and computer-assisted surgery. However, in the current clinical practice, radiologists still manually delineate the liver on each CT slice in order to achieve the most accurate segmentation results, but this manual process is quite time consuming, tedious, and laborious and also leads to significant intraobserver differences. In addition, liver segmentation is a challenging task due to the boundary blurring, low contrast, and uneven strength in liver CT images. Therefore, over the past decade, numerous studies have demonstrated effective, robust, and accurate algorithms (with varying degrees of success) for liver image segmentation in clinical practice. Depending on whether user interaction is required for liver segmentation, these methods can be broadly divided into two categories: automatic and semiautomatic methods [23].

Moreover, the use of automatic segmentation in clinical applications requires evaluating and comparing the accuracies of different segmentation models. Recently, deep learning approaches have been employed to automatically obtain the most suitable segmentation model from given training data. Also, remarkable performance outcomes were achieved by these approaches through creating multiple levels of abstraction and descriptive embedding in a hierarchy of increasingly complex features [24]. For example, a semisupervised CNN was designed by Liu et al. to significantly decrease the requirement for labelled training data [25]. Multiple sparse regression models (such as deep ensemble sparse regression networks) were employed by Suk and Shen for clinical decision-making tasks [26]. A parameter-efficient CNN was designed by Spasov et al. for performing 3D separable convolution, combining specific layers and dual learning, and hence predicting the transition from mild cognitive impairment (MCI) to Alzheimer's disease within 3 years [27]. A semisupervised graph convolutional network was trained by Parisot et al. on node subsets labelled with diagnostic outcomes for representing and processing sparse clinical data [28]. For five organ structures, Lustberg et al. compared automated atlas-based contour generation results obtained using a commercial deep learning module [29]. In addition, Ahn et al. used a fusion-based U-Net model for medical image segmentation. This model was employed to evaluate the clinical feasibility of an open-source deep-learning framework trained on the data of 70 patients with liver cancer and also compare the performance of this framework with that of another commercially available atlas-based automatic segmentation framework [30].

In our work, we further explore the combination of Mask R-CNN and GANs to enhance the pixel-wise classification performance. Similar approaches have been proposed in earlier studies. For example, Frid-Adar et al. [31] generated synthetic medical images using generative adversarial networks (GANs) and used the generated images to improve the CNN-based classification performance for medical images. In order to boost the lung nodule detection sensitivity in CT images, Han et al. [32] naturally placed realistic diverse lung nodules in CT images using a 3D multiconditional GAN (MCGAN). The 3D CNN-based detection outcomes were of higher sensitivity under any nodule size or attenuation at a fixed FPR. Indeed, the medical data scarcity was overcome by the MCGAN-generated lung nodules.

Furthermore, unsupervised learning is also widely used in image segmentation. For example, an intelligent framework was proposed by Rundo et al. [33], where robust tools for validating radiomics biomarkers were provided for seamless integration into clinical research environments. In particular, this framework optimized the segmentation for each individual image while also taking into account prior domain knowledge for the typical densities of candidate subregions. The automation of this approach allows for easy deployment in clinical research environments, without the need for any training data. Anter and Hassenian [34] proposed an improved approach for liver segmentation in CT images based on a fast fuzzy *c*-means clustering algorithm (FFCM), neutrosophic sets (NS), and a watershed algorithm. In order to increase the CT image contrast and remove high frequencies, histogram equalization and median filtering were used. An unsupervised medical anomaly detection GAN (MAD-GAN) method was proposed by Han et al. [35]. In this novel two-step method, the GANs are used to reconstruct multiple adjacent magnetic resonance imaging (MRI) slices of the brain, and then, brain diseases are diagnosed and staged based on multisequence structural MRI data. Also, Nakao [36] proposed an unsupervised anomaly detection method based on variational autoencoders (VAE-GAN) and demonstrated its ability to detect various lesions using a large chest radiograph dataset. Unlike the widely used supervised methods for computer-aided diagnosis or detection in chest radiographs, the VAE-GAN-based unsupervised method can detect lesions of any type and does not require any abnormal image samples or lesion labels for training.

## 3. Materials and Methods

### 3.1. The Original Mask R-CNN

The Mask R-CNN architecture is an enhanced variant of the R-CNN, Fast R-CNN [37], and Faster R-CNN [38] architectures. In particular, the Mask R-CNN was immediately preceded by the Faster R-CNN. The Mask R-CNN represents a general conceptually simple and flexible framework for object detection and segmentation, where high-quality segmentation masks are simultaneously generated for image instances. While the Faster R-CNN has two branches for classification and bounding box regression, the Mask R-CNN has an additional third branch for segmentation mask prediction on each region of interest (RoI). This mask branch is just a small fully convolutional network (FCN) which acts upon each RoI to perform pixel-wise segmentation mask prediction. The Mask R-CNN can be easily trained and incurs a small computational overhead in comparison to the Faster R-CNN.

**Figure 1:**
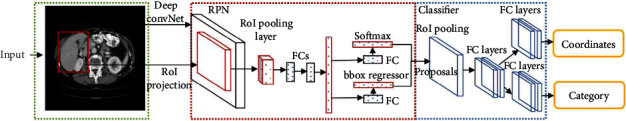
Framework of Mask R-CNN.

Figure 1 outlines the conventional Mask R-CNN framework for image segmentation. While the Mask R-CNN and Faster R-CNN have similar workflows, they still have some key differences. On the one hand, the Faster R-CNN suffers from spatial information loss and hence exhibits less accurate feature extraction and RoI detection. On the other hand, the Mask R-CNN uses a region proposal network (RPN) for feature extraction, as well as bounding box tight localization and classification. Also, the Mask R-CNN employs the RolPool method in feature extraction, RoI quantification, and handling of multiscale RoI features through maximum convergence. Moreover, the Mask R-CNN replaces the RolPool layer of the Faster R-CNN with an RoI alignment (RoIAlign) layer for the mask-labelled object area.

The Mask R-CNN can be employed for multitask learning, with the following loss function formulation:

(1) $$\begin{array}{c} L=L_{\text{cls}}+L_{\text{bbox}}+L_{\text{mask}}, \end{array}$$

where *L*_cls_ is the target classification loss, *L*_bbox_ is the regression loss for the target bounding box, and *L*_mask_ is the target segmentation loss, which is defined based on the target segmentation requirements in comparison with the traditional detection network.

### 3.2. Improved Mask R-CNN

**Figure 2:**
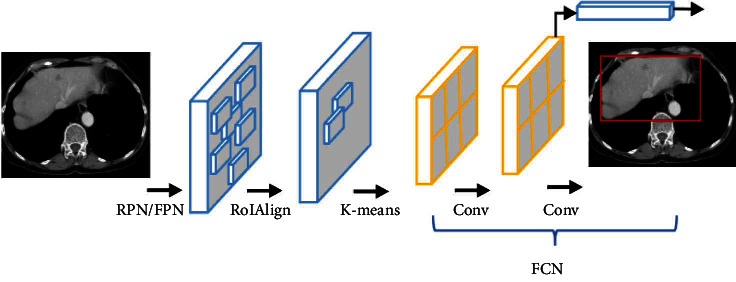
Framework of RoIAlign with *k*-means clustering.

With additional training iterations, the Mask R-CNN learns the global features of liver images, while the prediction box parameters are iteratively adjusted until they are really close to the true box parameters. In this paper, we strive to accelerate the convergence and improve the localization precision for liver detection and segmentation. We achieve these goals by analyzing the distribution of the aspect ratios of the liver images via *k*-means clustering. In the network training stage, as more training iterations are performed, the network learns the global liver characteristics in the CT images; the prediction box parameters are progressively adjusted; and finally, the ground-truth boxes are approached. In order to accelerate the convergence speed and improve the liver localization accuracy, the liver height and width characteristics are analyzed in the CT images, and hence, *k*-means clustering is applied to the height and width data using the Euclidean distance. This clustering algorithm measures the distance between patterns using the Euclidean distance and identifies the cluster centers through a given bounding box of anchors, where the output box is chosen as the closest one to an anchor. This process is repeated until the anchors reach a prespecified number.  Figure 2 shows the framework of RoIAlign with *k*-means clustering.

### 3.3. Generative Adversarial Networks

**Figure 3:**
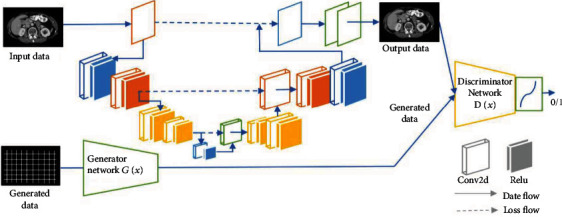
Framework of GAN Mask R-CNN.

The generative adversarial networks (GANs) were initially proposed by Goodfellow et al. in 2014. A generative model *G* captures the distribution of data, while a discriminative model *D* estimates the probability that a data sample came from the training samples rather than from *G*. The discriminative model *D* is trained to maximize the probability that both the training samples and the samples obtained from *G* are correctly labelled.  Figure 3 shows the proposed GAN Mask R-CNN architecture for liver image segmentation.

When the noise *z* is sampled from the latent space and fed into the generative model *G*, a sample *x* = *G* (*z*) is generated. For neural networks, the probability distribution *P*_*G*_ (*x*) of the generated samples might be significantly more complicated. The generative model *G* is trained to make the probability distributions *P*_*G*_ (*x*) and *P*_data_ (*x*) as close to each other as possible. The generative model *G* seeks to generate fake data to confuse the discriminative model (*D*), while the discriminator *D* seeks to differentiate between the real and fake data samples. This adversarial learning enforces the distribution generated by *G* to gradually approach the real data distribution.

The adversarial learning scheme can be formulated as the following optimization problem:

(2) $$\begin{array}{c} G=\arg\min_{G}\text{Div}\left(P_{G}\left(x\right),P_{\text{Data}}\left(x\right)\right), \end{array}$$

where Div denotes the divergence or dissimilarity between *P*_Data_(*x*) and *P*_*G*_(*x*). The function of the discriminator *D* can be mathematically defined as

(3) $$\begin{array}{c} D=\arg\max_{D}V\left(G,D\right), \end{array}$$

where the two models *D* and *G* interact through a two-player minimax competitive game with the objective function *V* (*G*, *D*):

(4) $$\begin{array}{c} V\left(G,D\right)=E_{x\sim P_{\text{data}}}\left[\log D\left(x\right)\right]+E_{x\sim P\left(G\right)}\left[\log\left(1-D\left(G\left(z\right)\right)\right)\right] \end{array}$$

Thus, the GAN parameter optimization problem can be formulated as

(5) $$\begin{array}{c} G=\arg\min_{G}\max_{D}V\left(G,D\right). \end{array}$$

The two models *G* and *D* are updated through alternating optimization. As the adversarial learning process evolves, the model *G* will generate data that gradually resembles the real data.

The training algorithm workflow is shown in Box 1.

Use the momentum learning rule (or any other standard rule) for the gradient-based updates.

## 4. Experimental Evaluation

We describe here the experiments we performed for evaluating the performance of our proposed GAN Mask R-CNN method. Also, we report and analyse the results of comparing our method against several state-of-the-art methods: FCN-8s [39], U-Net [40], 2D-FCN2 [41], 2D-FCN1 [42], 2D-dense-FCN [43], 3D-FCN [44], H-DenseUNet [45], 3D U-Net [46], IU-Net, and GIU-Net [47]. The comparisons were carried out in terms of standard evaluation metrics.

### 4.1. Experimental Setup and Data Collection

In our experiments, we used a CentOS7 system with an Intel Core i7 CPU with 128 GB memory and an NVIDIA RTX8000 48 GB GPU. We used Python 3.6 to implement the corresponding algorithms. Experiments were performed on the Codalab dataset (https://competitions.codalab.org/) [48, 49]. We employed 378 CT images for model training and the remaining sequences for testing. Codalab has liver CT images with three-phase ground-truth data. We used the enhanced CT format with a 512 × 512 image resolution.

### 4.2. Performance Evaluation with DSC and MICCAI Metrics

We use the DSC to assess the performance of the compared methods of liver image segmentation [50]. The DSC metric represents the spatial coincidence degree between the output and ground-truth segmentation results. Also, we consider five other segmentation metrics provided by the Society of Medical Image Computing and Computer-Assisted Intervention (MICCAI) [51, 52], namely, volume overlap error (VOE), average symmetric surface distance (ASSD), root-mean-square symmetric surface distance (RMSD), maximum symmetric surface distance (MSSD), and relative volume difference (RVD). The aforementioned segmentation metrics are mathematically defined as follows.

First of all, the DSC metric can be defined as

(6) $$\begin{array}{c} D\text{SC }\left(U_{1},U_{2}\right)=\frac{2\;|\;U_{1}\cap U_{2}\;|\;}{\left|U_{1}\right|+\left|U_{2}\right|}, \end{array}$$

where *U*_1_ and *U*_2_ denote corresponding output and ground-truth segmentation results, respectively. A higher DSC value indicates better segmentation performance. The DSC value ranges between zero (indicating a total dissimilarity between the output and ground-truth segmentation results) and one (indicating ideal total agreement between the output and ground-truth segmentation results).

The other five MICCAI metrics are defined as follows. Once more, the symbols *U*_1_ and *U*_2_ denote corresponding output and ground-truth segmentation results, respectively. Also, *S*(*U*_1_) and *S*(*U*_2_) denote the outlines of the segmented liver region and the associated ground-truth region, respectively. In addition, *d* (*v*, *S* (*U*_1_)) denotes the shortest distance between any image pixel *v* and *S*(*U*_1_). That is, *d*(*v*, *S*(*U*_1_)) = min_*SU*_1_∈*S*(*U*_1_)_ ‖*v* − *S*_*U*_1__‖, where ‖•‖ is the Euclidean distance operator. Apart from the DSC, lower values of four MICCAI metrics (VOE, ASSD, RMSD, and MSSD) indicate better matching between the output and ground-truth segmentation results. The RVD absolute value should be used in segmentation performance evaluation, since this metric might take negative values in the case of undersegmentation. The smaller the RVD absolute value is, the better the segmentation performance is. In general, the five MICCAI metrics are 0 when the segmentation is perfect.

(7) $$\begin{array}{c} \text{VOE}\left(U_{1},U_{2}\right)=100\times \left(1-\frac{\left|U_{1}⋂U_{2}\right|}{\left|U_{1}⋃U_{2}\right|}\right),\\ \text{RVD }\left(U_{1},U_{2}\right)=100\times \left(\frac{\left|U_{1}\right|-\;|\;U_{2}\;|\;}{\;|\;U_{2}\;|\;}\right),\\ \text{ASSD }\left(U_{1},U_{2}\right)=\frac{1}{\left|S\left(U_{1}\right)\right|+\left|S\left(U_{2}\right)\right|}\left(\underset{S_{U_{1}\in }S\left(U_{1}\right)}{\sum }d\;\left(S_{U_{1}},S\left(U_{2}\right)\right)+\underset{S_{U_{2}}\in S\left(U_{2}\right)}{\sum }d\left(S_{U_{2},}S\left(U_{1}\right)\right)\right),\\ \text{RMSD }\left(U_{1},U_{2}\right)=\sqrt{\frac{1}{\;|\;S\left(U_{1}\right)\;|\;+\;|\;S\left(U_{2}\right)\;|\;}}\times \sqrt{\underset{S_{U_{1}}\epsilon \text{ S}\left(U_{1}\right)}{\sum }d^{2}\left(S_{U_{1}}+S\left(U_{2}\right)\right)+\underset{S_{U_{2}}\in S\left(U_{2}\right)}{\sum }d^{2}\left(S_{U_{2},}S\left(U_{1}\right)\right)},\\ \text{MSSD}\left(U_{1},U_{2}\right)=\max\left\{\max_{{\text{S}}_{U_{1}}\epsilon \;S\;\left(U_{1}\right)}d\left(S_{U_{1}},S\left(U_{2}\right)\right),\max_{{\text{S}}_{U_{2}}\in S\left(U_{2}\right)}\;d\left(S_{U_{2}},S\left(U_{1}\right)\right)\right\}. \end{array}$$

### 4.3. Performance Evaluation with Binary Classification Metrics

Several other metrics were exploited as well for evaluating the performance of liver segmentation algorithms, where image segmentation was cast as a binary classification problem with positive and negative classes corresponding to the liver and nonliver image pixels, respectively [35]. The term TP (true positives) stands for the number of pixels that are claimed by the segmentation method to be positive, while they are really positive according to the ground-truth labelling. The term FP (false positives) depicts the number of pixels that are suggested to be positive by the segmentation method but are actually negative according to the ground-truth labelling. The term TN (true negatives) expresses the number of pixels claimed to be negative by the segmentation method, while they are actually negative according to the ground-truth labels. The term FN (false negatives) denotes the number of pixels claimed negative by the segmentation method but are actually positive according to the ground truth. Based on these terms, key evaluation indicators are defined as follows:

1. Overall accuracy: the ratio of the correctly labelled pixels to the total pixel count

(8) $$\begin{array}{c} \text{Accuracy}=\frac{\text{TN}+\text{TP}}{\text{TN}+\text{TP}+\text{FN}+\text{FP}} \end{array}$$

(2) Sensitivity: the ratio of the correctly detected liver pixels to all true liver pixels is called the sensitivity or the recall

(9) $$\begin{array}{c} \text{Sensitivity}=\frac{\text{TP}}{\text{TP}+\text{FN}} \end{array}$$

(3) Specificity: the ratio of the correctly detected background pixels to all true nonliver background pixels is called the specificity

(10) $$\begin{array}{c} \text{Specificity}=\frac{\text{TN}}{\text{TN}+\text{FP}} \end{array}$$

(4) Precision: the ratio of the correctly detected liver pixels relative to the total number of pixels labelled as liver pixels

(11) $$\begin{array}{c} \text{Precision}=\frac{\text{TP}}{\text{TP}+\text{FP}} \end{array}$$

(5) The false omission rate (FOR) of the liver pixels is defined as

(12) $$\begin{array}{c} \text{FOR}=\frac{\text{FN}}{\text{TN}+\text{FN}} \end{array}$$

(6) Based on the precision *P*, the false detection rate (FDR) can be defined as

(13) $$\begin{array}{c} \text{FDR}=1-\text{precision}=\frac{\text{FP}}{\text{TP}+\text{FP}} \end{array}$$

## 5. Analysis of the Experimental Results

**Figure 4:**
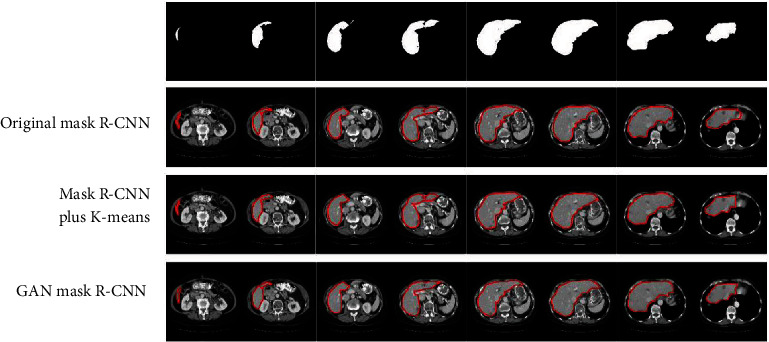
A comparison of the segmentation results between the improved and conventional Mask R-CNN algorithms.

Experiments were conducted to compare the performance of three schemes: the conventional Mask R-CNN, the Mask R-CNN with *k*-means clustering (for optimization of the fully connected layer parameters) [36], and the GAN Mask R-CNN which boosts the segmentation performance with adversarial learning capabilities. Eight slices of liver images (with normal and pathological cases) were collected and used to investigate the influence of the *k*-means clustering and GAN modules on the Mask R-CNN segmentation outputs.  Figure 4 gives a comparison of the results which are shown in red contours.

As shown in Figure 4, the conventional Mask R-CNN method obviously missed areas of marginal liver regions during liver slice processing and hence resulted in segmentation errors. The Mask R-CNN with *k-*means clustering managed to correct these segmentation errors by incorporating the aspect ratio information of the liver image sequence. However, there are still visible segmentation errors in marginal liver regions. Our proposed GAN Mask R-CNN improved, to a certain extent, the segmentation accuracy and robustness for each slice in the sequence. This is an obvious advantage of our proposed scheme over the two other Mask R-CNN variants.

As shown in Table 1, the experimental results of the GAN Mask R-CNN were evaluated in terms of the following indicators: the overall accuracy, sensitivity, specificity, precision, FOR, and FDR. Our proposed GAN-based algorithm has a relatively high evaluation accuracy, as well as very low omission rate. This performance can be ascribed to the training adequacy and the relative robustness of the output boundary, though missegmentation or oversegmentation errors still exist.

Table 2 shows that the GAN Mask R-CNN performs significantly better (95.3%) than the other two algorithms. Two exceptions are the VOE result of the conventional Mask R-CNN (21.58%) and the MSSD result of the Mask R-CNN with *k*-means clustering (21.32%). In addition, it is clear that the GAN module significantly enhances the segmentation performance according to the other indicators.

**Figure 5:**
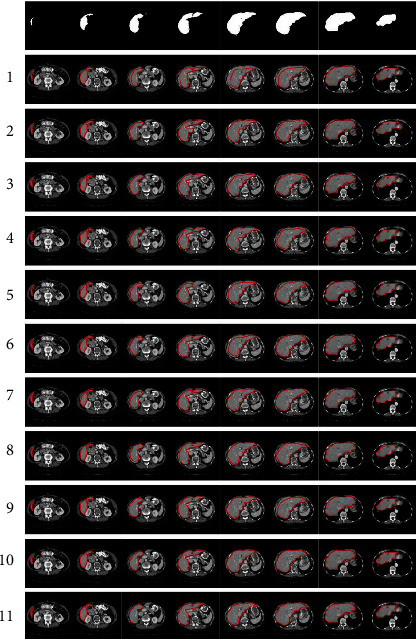
A comparison of the liver segmentation results between three algorithms: (1) FCN-8s, (2) U-Net, (3) 2D-FCN2, (4) 2D-FCN1, (5) 2D-dense-FCN, (6) 3D-FCN, (7) H-DenseUNet, (8) 3D U-Net, (9) IU-Net, (10) GIU-Net, and (11) GAN Mask R-CNN.

Further experiments were made to assess the impact of the GANs and *k*-means clustering modules on improving the segmentation outcomes in comparison to the FCN-8s, U-Net, 2D-FCN2, 2D-FCN1, 2D-dense-FCN, 3D-FCN, H-DenseUNet, 3D U-Net, IU-Net, and GIU-Net algorithms. The performance was evaluated using the metrics of accuracy, recall, specificity, precision, FOR, and FDR. A comparison of the segmentation results of the ten algorithms is shown in Figure 5. The test data for this comparison includes slices with large, medium, and small liver regions.

The results in Figure 5 show that for the six liver slices considered, some undersegmentation or oversegmentation errors are made by the FCN-8s, U-Net, 2D-FCN2, 2D-FCN1, 2D-dense-FCN, 3D-FCN, H-DenseUNet, 3D U-Net, IU-Net, and GIU-Net algorithms. Also, some of the segmented liver slices do not exhibit complete boundaries, while others have extraneous parts that do not belong to the original liver slices. However, our GAN Mask R-CNN method can produce more solid boundaries and return segmented liver slices with no extra holes.

Table 3 indicates that the GAN Mask R-CNN method significantly outperforms the other algorithms, except for the GIU-Net method which shows a better specificity, as well as the IU-Net method which shows a better precision compared to our method. Anyway, our algorithm clearly outperforms the IU-Net and GIU-Net algorithms according to all other indicators. In addition, our algorithm shows superior performance on all six indicators in comparison to the FCN-8s, U-Net, 2D-FCN2, 2D-FCN1, 2D-dense-FCN, 3D-FCN, H-DenseUNet, and 3D U-Net algorithms.

## 6. Conclusions

This paper introduces a new method for liver image segmentation in CT sequences where the GANs and the Mask R-CNN methods are combined. However, we found that most images exhibited noisy features in one way or another in the liver segmentation process [52, 53]. Under the influence of complex surrounding blood vessels and organs, the liver shape varies between different sections in the same set of CT images, and there are many soft tissues in the abdominal cavity with a density similar to that of the liver soft tissue [39]. Also, medical CT imaging often presents problems of low contrast and uneven grey-scale intensities, making it difficult to segment liver images accurately in the area of interest [18, 54–56].

In this work, we proposed a new CT-based liver image segmentation framework. Firstly, we sought to get more important anchors (and hence improve the segmentation results) through a *k*-means clustering algorithm which was used to lock the image aspect ratio and reduce redundant and useless anchors. Secondly, we addressed the problem of the presence of noisy features in liver images, with no image enhancement typically applied, rendering a large number of images unusable and reducing the segmentation accuracy. Specifically, we employed a GAN architecture into our segmentation framework and demonstrated good performance in terms of six indicators: DSC, VOE, RVD, ASSD, RMSD, and MSSD. Thirdly, we compared our framework with that of FCN-8s, U-Net, 2D-FCN2, 2D-FCN1, 2D-dense-FCN, 3D-FCN, H-DenseUNet, 3D U-Net, IU-Net, and GIU-Net. This comparison was based on six indicators: overall accuracy, sensitivity, specificity, precision, FOR, and FDR. Our improved GAN Mask R-CNN architecture demonstrated the best overall performance. We hope that our work can help radiology practitioners to further improve the diagnosis, timely detection, and treatment of liver diseases and also reduce the risk of death due to liver cancer.

In many studies, open-source deep learning tools may be applied for automatic liver segmentation. The performance outcomes of such tools could be compared with the outcomes of conventional knowledge-based planning tools, which typically yield acceptable accuracy levels as well as good reproducibility for clinical use. Additionally, patient-specific dose prediction improves the efficiency and quality of radiation treatment planning. In particular, this prediction can significantly reduce the treatment planning time. In the future, we envisage that deep-learning-based automatic segmentation will become clinically useful, especially for dynamic daily treatment plans based on multimodality imaging.

## Figures and Tables

**Box 1:**
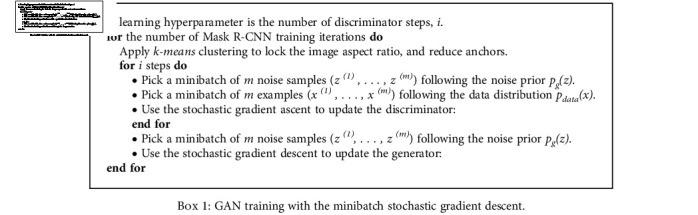
GAN training with the minibatch stochastic gradient descent.

**Table 1:** Comparison of segmentation algorithms before and after enhancements.

Algorithm type	Accuracy (%)	Recall (%)	Specificity (%)	Precision (%)	FOR (%)	FDR (%)
Original Mask R-CNN	85.2	88.3	89.0	87.4	20.1	12.6
Mask R − CNN + *k* − means	86.4	88.2	89.1	87.5	19.2	12.5
GAN Mask R-CNN	91.3	92.2	92.4	92.3	13.1	7.7

**Table 2:** Comparison of segmentation algorithms before and after enhancements.

Algorithm type	DSC (%)	VOE (%)	RVD (%)	ASSD (mm)	RMSD (mm)	MSSD (mm)
Original Mask R-CNN	92.4	9.32	0.53	3.32	5.42	20.67
Mask R − CNN + *k* − means	93.3	9.11	0.54	2.23	3.32	21.32
Improved Mask R-CNN	95.3	9.34	0.44	2.23	2.32	21.58

**Table 3:** A comparison of four liver segmentation algorithms based on six metrics.

No.	Algorithm type	Accuracy (%)	Recall (%)	Specificity (%)	Precision (%)	FOR (%)	FDR (%)
1	FCN-8s	73.2	75.3	75.0	74.4	20.1	25.6
2	U-Net	71.3	73.2	74.1	75.5	19.2	24.5
3	2D-FCN2	75.4	77.2	78.4	76.3	13.1	23.7
4	2D-FCN1	72.3	73.4	73.6	74.5	14.5	25.5
5	2D-dense-FCN	75.3	76.7	75.6	77.8	12.9	22.2
6	3D-FCN	82.3	81.7	82.9	80.1	10.1	19.9
7	H-DenseUNet	83.3	83.4	83.0	80.9	10.0	19.1
8	3D U-Net	86.3	87.7	88.3	87.3	11.5	12.7
9	IU-Net	88.3	89.3	90.2	92.9	16.3	7.1
10	GIU-Net	90.2	91.6	92.8	90.7	9.8	9.3
11	GAN Mask R-CNN	91.3	92.2	92.4	92.3	13.1	7.7

## Data Availability

Data access is available on request through the Codalab competition website (https://competitions.codalab.org/). The contact person is Xiaoqin Wei, School of Medical Imaging, North Sichuan Medical College, China, E-mail address: xiaoqin_wei_nsmc@163.com.
